# The effects of mental health problems in childhood and adolescence in young adults: Results of the KiGGS cohort

**DOI:** 10.25646/8863

**Published:** 2021-12-08

**Authors:** Robert Schlack, Nele Peerenboom, Laura Neuperdt, Stephan Junker, Ann-Kristin Beyer

**Affiliations:** 1 Robert Koch Institute, Berlin Department of Epidemiology and Health Monitoring; 2 University of Cambridge, Department of Psychiatry

**Keywords:** INTERNALISING, EXTERNALISING, YOUNG ADULTS, DEVELOPMENT OUTCOMES, POPULATION-BASED

## Abstract

Mental health problems in childhood and adolescence may have effects into adulthood. With the KiGGS cohort, data are available for the first time that can be used to track the effects of internalising and externalising problems in childhood or adolescence into young adulthood on a national database. From the KiGGS baseline survey (2003–2006) to KiGGS Wave 2 (2014–2017), a total of 3,546 children and adolescents aged 11 to 17 years were tracked over a period of eleven years into young adulthood. Mental health problems in childhood or adolescence were variously associated with impaired mental health, lower life satisfaction and poorer quality of life and indicators of sexual and reproductive health in young adulthood.

When psychosocial protective factors at the time of the KiGGS baseline survey were considered, the longitudinal correlations of internalising and externalising problems with indicators of mental health, life satisfaction and physical and psychological quality of life decreased, as did, to a lesser extent, the correlations with indicators of sexual and reproductive health and, for externalising disorders, also with low educational status (reference: medium). Implications for prevention and intervention are discussed.

## 1. Introduction

Mental health problems affect the developmental opportunities of children and adolescents and may have effects into adulthood [1–3]. More than half of all mental disorders in adulthood begin in childhood or adolescence [2, 4, 5]. However, not only children with diagnosed mental disorders may present with mental health problems later in life [6]. Also, children and adolescents with symptoms that do not fully meet the criteria for the diagnosis of a mental disorder are at increased risk for impaired mental health in adulthood [3].

Previous studies have shown that internalising and externalising problems (Info box) in childhood and adolescence can be associated with various effects in adulthood regarding the mental health, quality of life or academic achievement [9–13]. For example, subjects with internalising disorders in childhood or adolescence are more likely to display symptoms of anxiety or depression and impaired health-related quality of life as adults [9, 10, 12, 14, 15]. On the other hand, children and adolescents with externalising problems often have lower educational success than their unaffected peers and are at an increased risk of later use of psychoactive substances in later life [9, 10].

In the research of resilience, protective factors for the mental health have been discussed since the late 1950s [16]. Psychosocial resources at the individual or environmental level (i.e., family and social level) that contribute to mitigating developmental risks of children and adolescents or to maintaining and protecting their mental health can be described as protective or compensatory factors [16, 17]. Children and adolescents who develop into socially successful adults despite existing developmental risks such as poverty, experiences of violence, family discord, parental divorce, parental psychopathology, physical illnesses or disabilities are referred to as resilient [16, 18]. However, long-term studies on a nationally representative level still remain rare.

For the first time, the German Health Interview and Examination Survey for Children and Adolescents (KiGGS) provides, on a national level, a population-based longitudinal data set, which can be used to track individuals with internalising and externalising problems over a period of eleven years, from childhood or adolescence into young adulthood.

The first research question of this contribution adresses the relationship between mental health problems in childhood or adolescence and developmental outcomes in young adulthood as operationalized in the KiGGS study. These include aspects of mental health, life satisfaction and health-related quality of life, latest educational status, partnership status as well as aspects of sexual and reproductive health. The second research question investigates to what extent the availability of personal, family and social protective resources in children and adolescents as captured in the KiGGS study [19] is related to developmental outcomes in young adulthood in subjects with childhood internalising and externalising problems.

**Info box**

**Internalising and externalising problems**

Mental health problems in childhood and adolescence include emotional and behavioural problems. Common internalising (i.e., more inwardly directed) problems are, for instance, anxiety, shyness, experiences of rejection, brooding, frequent worrying or frequent crying and peer problems. More outwardly directed, expansive behaviours such as motor restlessness, a high degree of distractibility, attention problems, frequent interrupting and disturbing others or aggressive and dissocial or rule-breaking behaviour up to and including delinquency are referred to as externalising problems [7, 8].

## 2. Methods

### 2.1 Sample design and study procedure

**Figure 1:**
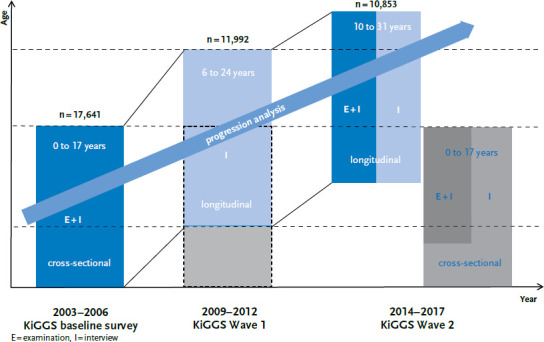
Structure of the German Health Interview and Examination Survey for Children and Adolescents (KiGGS) Source: Mauz et al. 2017 [21]

Within the framework of the KiGGS cohort, the children and adolescents who were first examined and interviewed in the baseline survey in the years 2003–2006 are followed up. The KiGGS baseline survey has so far been followed by two further survey waves, KiGGS Wave 1 (2009–2012) and KiGGS Wave 2 (2014–2017) at intervals of six and eleven years, respectively [20] (Figure 1). The present analyses are based on two survey periods, the KiGGS baseline survey and KiGGS Wave 2.

#### The KiGGS baseline survey

The KiGGS baseline survey was conducted in 167 representatively selected cities and municipalities in Germany with a total of 17,641 girls and boys aged 0 to 17 years. It was the first nationally representative, population-based survey on child and adolescent health in Germany and included physical, mental, and social aspects of health as well as aspects of the health behavior of children and adolescents. The children were physically examined and tested and the parents filled in written questionnaires about the health of their offspring. From the age of eleven on the children and adolescents were also interviewed themselves [22, 23].

#### KiGGS Wave 2

The second follow-up survey, KiGGS Wave 2 (2014–2017), was conducted as an examination and survey, too [21]. In addition to a newly drawn cross-sectional sample for the age cohorts from 0 to 17 years in the 167 study sites [24], all participants of the KiGGS baseline survey who were still available and willing to participate again were invited to a written survey and – if they still lived in the study sites – to the examination component. In the meantime, they were 10 to 31 years old. A total of 10,853 longitudinal participants (KiGGS cohort) aged 10 to 31 years could be re-interviewed at the time of KiGGS Wave 2 this way [25]; the total re-participation rate was 62% [26].

### 2.2 Instruments and indicators

#### KiGGS baseline survey

Mental health problems were assessed with the Strengths and Difficulties Questionnaire (SDQ) in the parent rated version. The SDQ captures the most important areas of mental health problems in childhood and adolescence based on four so-called problem scales (1) ‘Emotional problems’ (item contents e.g. ‘Many worries; often seems worried’, ‘Many fears; easily scared’); (2) ‘Behavioural problems’ (item contents e.g. ‘Often has temper tantrums or hot tempers’, ‘Often lies or cheats’); (3) ‘Hyperactivity problems’ (item contents e.g. ‘Constantly fidgeting or squirming’, ‘Easily distracted, concentration wanders’); and (4) ‘Peer problems’ (item contents e.g. ‘Picked on or bullied by other children’, ‘Has at least one good friend’) with five items each [27]. In addition, the SDQ contains the strengths scale ‘Prosocial Behaviour’, which, however, is not considered here. Using normative data, participants can be classified as ‘normal’, ‘borderline’ and ‘abnormal’ (see [28]). For the present paper, participants with ’borderline’ or ‘abnormal’ scores on the subscales ‘emotional problems’ or ‘peer problems’ were combined into a subgroup with predominantly internalising problems. Participants with ‘borderline’ or ‘abnormal’ scores on the subscales ‘behavioural problems’ or ‘hyperactivity problems’ were combined into a subgroup with predominantly externalising problems [29, 30].

Personal resources were assessed applying a scale comprising three items from the General Self-Efficacy Scale WIRKALL (e.g. ‘I can usually handle whatever comes my way.’) [31, 32] and one item each from the Bern Questionnaire for Optimism (‘My future looks bright.’) [33] and the Sense of Coherence Scale (‘The things I do every day give me pleasure and are fun.’) [34]. Answers were given by children and adolescents aged eleven and older.

Family cohesion was assessed using a modified version of the Schneewind, Beckmann and Hecht-Jackl family climate scale (item contents e.g. ‘In our family, everyone is responsive to everybody else’s worries and needs’, ‘We all really get along well with each other’) [35]. The nine items were answered by children and adolescents aged 11 to 17.

Social support was measured with a modified version of Donald and Ware’s Social Support Scale [36] with a total of eight items. The items were answered by children and adolescents aged eleven and older.

#### KiGGS Wave 2

The five item Mental Health Inventory (MHI-5) was used to assess overall mental health. It assesses the frequency of various emotions over the past four weeks [37]. The eight-item version of the Patient Health Questionnaire (PHQ) was used to capture symptoms of depression during the last two weeks [38–40]. Panic disorder symptoms in the past four weeks were assessed with the five item PHQ-Panic screener [40]. Eating disorder symptoms were assessed with the SCOFF (Sick, Control, One, Fat, Food) questionnaire [41]. This questionnaire comprises five items related to core symptoms of anorexia and bulimia nervosa. Health-related quality of life was assessed with the German version of the eight-item Short-Form Health Survey (SF-8) [42] with two subscales pertaining to mental and physical health-related quality of life. General life satisfaction was measured using the Personal Wellbeing Index (PWI-A) [43]. The PWI-A consists of seven items on standard of living, health, personal relationships, security, belonging to a community, and security in the future.

The educational status of young adults aged 18 years and older was captured using the ISCED-11 (International Standard Classification of Education) [44], which allows classification into the educational categories low, medium and high.

Smoking and risky alcohol consumption were used as indicators of substance use. Smoking was recorded with a binary yes/no question. Occasional smokers were classified as smokers. Risky levels of alcohol consumption were assessed with the AUDIT-C (Alcohol use disorders identification test – consumption) [45]. The AUDIT-C is the short version of the AUDIT screening questionnaire and allows the calculation of a sum score for risky alcohol consumption based on three items.

Regarding partnership and sexual and reproductive health, participants were asked whether they were currently in a stable partnership. In addition, they were asked about their age at first sexual intercourse and the number of sexual partners. Condom use was assessed with the question ‘Do you use condoms during sexual intercourse?’ with three possible answers offered (‘Yes, always’, ‘Yes, occasionally’, ‘No’). All adult participants were asked whether they had biological children and whether they were planned or unplanned.

### 2.3 Statistical methods

To analyse the effects of internalising and externalising problems in childhood or adolescence on developmental outcomes in young adulthood, logistical and linear regression models were specified depending on the discrete or continuous character of the dependent variable. The developmental outcomes in young adulthood served as dependent variables and internalising and externalising problems as independent variables.

In a first model, the outcomes in young adulthood (at KiGGS Wave 2) were predicted by internalising and externalising problems in childhood or adolescence (at KiGGS baseline survey) and controlled for age (in years), gender, migration background and socioeconomic status (SES). In a second model, the protective factor scales ‘personal resources’, ‘family cohesion’ and ‘social support’, (at KiGGS baseline survey), were also included. The models for risky alcohol consumption, condom use and contraception were additionally adjusted with a quadratic age term and the models for condom use and contraception moreover adjusted for stable partnership and number of sexual partners. Differences between groups with a p-value <0.05 were considered statistically significant.

All analyses were conducted with Stata (version 15.1) using survey procedures and longitudinal weights to compensate for study design and putative sampling bias due to selective re-participation.

## 3. Results

### 3.1 Sample description

The present analyses are based on data from a total of 3,546 21- to 31-year-old participants (average age 25.0 years): 55.4% were female and 44.6% were male. The proportion of participants with low SES was 10.0%, with medium SES 61.5%, and with high SES 28.4%. The proportion of participants with a migration background of both parents was 9.3%. A total of 22.6% of the sample was presenting with internalising (women: 22.9%, men: 22.1%) and 15.4% were presenting with externalising problems in childhood or adolescence (women: 12.8%, men: 18.6%).

### 3.2 Internalising problems

Participants with internalising problems in childhood or adolescence report poorer general mental health, more depressive symptoms and a higher likelihood of presenting eating disorder symptoms (Table 1). On average, they show lower overall life satisfaction and lower physical and psychological quality of life as young adults aged up to 31 years. Internalising problems are not significantly associated with academic achievement. Further, there is no increased probability of suffering from panic disorders in young adulthood. Similarly, no increased risk of smoking or risky alcohol use was found for those participants. The latter even appears to be less likely among participants with internalising problems in childhood or adolescence. The probability of being in a stable partnership in young adulthood is significantly lower for participants with internalising problems in childhood or adolescence than for participants without those problems. Furthermore, these individuals show a higher age at first sexual intercourse and an increased likelihood of having unplanned children.

#### Effects of protective factors in childhood or adolescence: internalising problems

When including personal, family and social protective factors at KiGGS baseline in the model, the coefficients for internalising problems in childhood or adolescence decrease in the models for general mental health, depressive symptoms and eating disorder symptoms in young adults. This is also true for the models of general life satisfaction and physical and psychological quality of life in adults. With regard to educational status and smoking or alcohol consumption, no or only very slight effects were found. The relationship between internalising problems in childhood or adolescence and a lower probability of living in a stable partnership in young adulthood is not significant after adjusting for the protective factor scales. When the protective factor scales are considered, the age of first sexual intercourse increases as does the probability of having unplanned children.

### 3.3 Externalising problems

Externalising problems in childhood or adolescence are associated with poorer general mental health, higher incidence of depressive symptoms and an increased likelihood of suffering from eating disorder symptoms in young adulthood (Table 2). They are significantly associated with lower overall life satisfaction and lower physical health-related quality of life in young adulthood. No significant association with psychological health-related quality of life was found. Participants reporting externalising problems in childhood or adolescence have an increased probability of lower educational status and of being smokers in young adulthood. In contrast, no correlations with risky alcohol use are found. On average, participants with externalising problems in childhood or adolescence are younger at first sexual intercourse and have had a larger number of sexual partners. On the other hand, they are less likely to have unplanned children.

#### Effects of protective factors in childhood or adolescence: externalising problems

After adjusting for personal, family and social protective factors, the coefficient for externalising problems in childhood or adolescence decreases in the model for general mental health, depressive symptoms and eating disorder symptoms in young adulthood. The coefficient in the model for life satisfaction is no longer significant, the coefficient in the model for physical quality of life in young adulthood decreases in extent but remains significant. The coefficient for low versus medium educational status decreases slightly after adjustment with the protective factor scales. After adjustment with the protective factor scales, the probability of being a smoker in young adulthood decreases. The average age at first sexual intercourse remains unaffected, the coefficient in the model for the number of sexual partners is no longer significant, and the probability of having unplanned children decreases.

## 4. Discussion

According to the data of the KiGGS cohort, internalising and externalising problems in childhood or adolescence impact on the mental health, life satisfaction, health-related quality of life, academic achievement, risky health behaviour and sexual and reproductive health in young adulthood.

### Internalising problems

In this study, the mental health outcomes of participants with predominantly internalising problems in childhood or adolescence in young adulthood are often poorer and subjects report lower life satisfaction and decreased psychological and physical health-related quality of life. Comparable associations were also found in a longitudinal study in the US [15]. In that study, respondents who were presenting with internalising problems when they were five to twelve years old showed lower physical and psychological quality of life in young adulthood, too; they were less physically active and presented eating disorder symptoms more frequently. This was particularly true for young women [15]. Like in our study, in other international studies internalising problems in childhood and adolescence were shown to be stable into adulthood [14, 46]. For example, a US study with an observation period of 30 years showed temperament differences in infancy to be associated with internalising symptoms in adulthood: children who were shy or anxious at the age of 14 months were more introverted, had more social problems, and suffered more depressive and anxiety symptoms in adulthood [14]. In line with other studies [47], we did not find associations between internalising problems in childhood or adolescence and later academic achievement.

In addition, we did not find a higher likelihood for risky alcohol or tobacco consumption in young adulthood for participants with internalising problems in childhood or adolescence. According to our data, risky alcohol consumption is actually lower among those than among participants without such problems in childhood or adolescence. A longitudinal study from Australia came up with similar results, as children with internalising problems at the age of five were less likely to smoke in adolescence (at the age of 14 years) than non-affected children [49]. In a Finnish cohort study, the use of psychoactive substances such as alcohol, cannabis or other illicit drugs was not associated with internalising problems in childhood, too [50].

Additionally, the KiGGS data indicates that individuals with internalising problems in childhood or adolescence are less likely to be in a stable partnership in young adulthood. Also, participants with internalising problems in childhood or adolescence tend to become sexually active at a later point in life than the non-affected. They display, however, an increased likelihood of having unplanned children. Several studies have shown associations between internalising problems and early parenthood, both for young mothers and young fathers [51, 52]. However, there are gender-specific aspects concerning the association between early internalising problems and sexual and reproductive health. For instance, boys with internalising problems at the age of eight to ten years tend to become sexually active at a younger age, whereas this association was not found in girls [54]. Further associations between internalising problems in childhood and adolescence with sexual and reproductive health have been previously described in research literature. A New Zealand longitudinal study found that internalising problems were associated with a lower likelihood of early first sexual intercourse and with a decreased likelihood of risky sexual behaviour in young adulthood, such as frequently changing sexual partners or not using condoms during sexual intercourse [55].

### Externalising problems

Externalising problems in childhood and adolescence have long been in the focus of developmental psychopathology research due to their considerable impact on the further course of life [56]. In line with our data, the literature shows that externalising problems in childhood and adolescence are associated with higher risks for depressive and anxiety symptom. Such associations hold also true with externalising disorders such as attention deficit/hyperactivity disorder (ADHD), substance use disorders or antisocial personality disorders [57, 58]. In line with our results, a Norwegian study showed that individuals with a high and stable trajectory of externalising problems from age 1.5 to 14.5 years displayed significantly lower life satisfaction in young adulthood [59]. Also in line with our data, the Dutch TRAILS study showed that externalising problems in childhood or adolescence were associated with lower educational success later in life [47]. In our study, children or adolescents with externalising problems had significantly higher risks of smoking in adulthood, but not for risky alcohol consumption. In contrast, the BELLA study, the in-depth mental health module of the KiGGS study, for which the data was collected in parallel to KiGGS Wave 1, the likelihood of risky alcohol consumption in young adulthood is 1.6 times higher for participants with externalising problems than for individuals without behavioural problems However, this discrepancy may be explained by age differences of the participants and different definitions of externalising problems. According to our data, the likelihood of becoming sexually active at an early age is significantly higher for children or adolescents with externalising problems. However, they were not less likely to be in a stable partnership in young adulthood than participants without such problems in childhood or adolescence. Comparable results were found in the previously mentioned New Zealand cohort study, where externalising problems in childhood were similarly associated with early sexual intercourse (before the age of 16), sexual intercourse without condom use and with more frequent changes of partners [54, 55]. For boys in particular, an early onset of externalising problems at the age of five was found to be predictive of an early onset of sexual activity (before the age of 16) [54].

### Effects of protective factors

The present study is among the few to examine the effects of protective factors on the relationship between internalising and externalising problems in childhood or adolescence and developmental outcomes in young adulthood on a national level. The present analyses show that the availability of psychosocial protective factors in childhood or adolescence impacts most on mental health, life satisfaction and health-related quality of life in young adulthood. This holds true for both children and adolescents with internalising and externalising problems. Even when controlling for important confounding factors such as age, gender, migration background, and socioeconomic status, the overall risk for poorer educational achievement in the presence of externalising problems in childhood or adolescence remains. To some – albeit small – extent, protective factors appear to mitigate the risks of low (versus medium) educational status for those with externalising problems. To a certain extent, a young adult person’s sexual and reproductive health also seems to be related to the available psychosocial protective resources in childhood and adolescence. For example, when adjusting for the protective factors young adult participants with internalising problems in childhood or adolescence displayed the same likelihood of being in a stable partnership as non-affected individuals in our study. However, protective factors did not impact on the increased likelihood of having unplanned children. If the protective factors were taken into account, the increase in number of sexual partners in the group of participants with externalising problems in childhood or adolescence became non-significant. These results highlight for both subjects with internalising and externalising problems potential starting points for sexual education interventions considering also psychosocial resources. Notably, the risk of having unplanned children appears lower among participants with externalising problems in childhood or adolescence, regardless of protective factors. A yet unpublished analysis on the basis of our data shows individuals diagnosed with ADHD in childhood or adolescence presenting with a higher probability of having unplanned children. This will require further in-depth analyses.

In our analyses, we considered personal, family and social protective factors in toto regardless of their respective dimensions. Specific analyses remain a task for future investigations.

It is known from other studies that protective factors have positive effects on later mental health and quality of life, both in the presence of internalising and externalising problems in childhood and adolescence. For example, a meta-analysis of 57 randomised controlled trials on the effects of interventions that focus on strengthening personal protective factors shows that strengthening the resilience of children and adolescents may have a protective effect regarding the development of later mental health problems. The development of internalising disorders in particular can apparently be significantly reduced by promoting personal protective factors during childhood and adolescence [60]. However, family and social protective factors also play an important role in mitigating risks to physical and mental health. In a US longitudinal study, family protective factors were found to be protective against the adverse effects of childhood violence on the development of externalising problems [61].

### Limitations and strengths

There are a number of limitations to the present analyses. For case number reasons in the group of the young adults, only the data from the first and third survey date of the KiGGS cohort were used. Despite the longitudinal character of the data, and not withstanding evidence for a causal effect, the results should therefore not be interpreted causally. Although the KiGGS cohort is based on the participants of the nationally representative KiGGS baseline survey it has to be kept in mind that a cohort’s representativeness decreases over time for various reasons: for example due to changes to the panel such as changes in marital status, the birth of children or changes in occupational and educational status, or due to panel mortality (i.e. changes in the composition of the sample due to the selective drop-out of participants). For instance, the proportion of participants with a low SES is 10% in the cohort sample and therefore only half as high as in the baseline sample. However, the analyses were weighted for drop-outs and additionally all analyses were controlled for SES. The present analyses provide a first overview of the correlates and the possible impacts of internalising and externalising problems in childhood or adolescence as well as the putative role of psychosocial protective factors. The statistical adjustment of the models with the protective factor scales does, however, not allow to evaluate whether the examined risks (internalising or externalising problems in childhood or adolescence) are actually buffered by the protective factors. Such thesis would require interaction analyses. However, the present results allow to conclude that the promotion of psychosocial protective factors in childhood and adolescence may be related to improvements to some of the developmental outcomes. Strengths of the present analyses include the longitudinal approach, the follow-up of participants over a period of eleven years, the large number of available indicators and the sample, which for the first time allowed to examine longitudinal data on the longterm development of children and adolescents with mental health problems at a national level in Germany.

## Conclusion

The present data shows that both internalising and externalising mental health problems in childhood or adolescence appear closely associated with the mental health, life satisfaction and the health-related quality of life as well as with the sexual and reproductive health in young adulthood. Beyond that, externalising problems appear associated with a lower educational status and an increased likelihood of smoking in adulthood. Mental health problems in childhood or adolescence may thus significantly impact the opportunities of enjoying a healthy and socially successful life of those affected. The results thus suggest that fostering protective factors in childhood and adolescence may be beneficial for youths presenting with mental health problems in order to mitigate multifaceted risks in young adulthood. The effects of psychosocial protective factors highlighted by the analyses point to possible opportunities for resource-based prevention and intervention. In-depth analyses on the potentially differential effects of the various protective factor dimensions (personal, familial and social) remain needed.

## Key statements

- Mental health problems during childhood or adolescence are associated with detrimental developmental outcomes in young adulthood.
- Children and adolescents with mental health problems often display impaired mental health, lower life satisfaction and poorer health-related quality of life as adults.
- Individuals with internalising problems in childhood or adolescence are, on average, older at first sexual intercourse and are more likely to have unplanned children.
- On Average, children and adolescents with externalising problems come up with lower academic success, tend to be smokers and to having had more sexual partners.
- The availability of psychosocial protective factors in childhood or adolescence may attenuate the effects of initial mental health problems with respect some adverse developmental outcomes in young adulthood.

## Figures and Tables

**Table 1:** Predicting developmental outcomes in young adulthood (age 21 to 31 years) in the presence of internalising problems in childhood or adolescence using linear and logistic regression models^1^ Source: KiGGS baseline survey (2003–2006), KiGGS Wave 2 (2014–2017)

Outcome in young adulthood^2^	Coefficient for internalising problems in Model 1^3^	Coefficient for internalising problems in Model 2^3^, ^4^
**Mental health**		
General mental health (MHI-5) (n=3,449)	B=-6.03^***^	B=-5.10^***^
Depressive symptoms (PHQ-8) (n=3,456)	B=1.35***	B=1.21***
Panic disorder (PHQ panic) (n=3,421)	OR=1.95	OR=2.11*
Eating disorder symptoms (SCOFF) (n=3,481)	OR=1.80***	OR=1.73***
**Life satisfaction and quality of life**		
General life satisfaction (PWI-A) (n=3,455)	B=-6.67^***^	B=-5.27^**^
Physical quality of life (SF-8) (n=3,480)	B=-1.06^**^	B=-0.82^*^
Psychological quality of life (SF-8) (n=3,480)	B=-3.11***	B=-2.68^***^
**Education status (ISCED)**		
Low vs. medium (n=3,463)	RRR=1.24	OR=1.17
High vs. medium (n=3,463)	RRR=0.85	OR=0.89
**Substance use**		
Risky alcohol consumption (Audit-C)^5^ (n=3,479)	B=-0.47^***^	B=-0.45^***^
Smoking (n=3,492)	OR=0.84	OR=0.87
**Partnership, sexual and reproductive health**		
Permanent partnership (n=3,491)	OR=0.79*	OR=0.83
Age of first sexual intercourse (n=3,192)	B=0.50^**^	B=0.38^**^
Number of sexual partners (n=3,206)	B=-0.05	B=-0.05
General vs. occasional condom use^5^, ^6^ (n=3,177)	RRR=1.01	OR=1.02
No vs. occasional condom use^5^, ^6^ (n=3,177)	RRR=0.91	OR=0.94
Risk for unplanned children (n=340)^7^	OR=2.24^*^	OR=2.40^**^

B = Beta coefficient, OR = Odds Ratio, RRR = Relative Risk Ratio, MHI = Mental Health Inventory, PHQ = Patient Health Questionnaire,

SCOFF = Sick, Control, One, Fat, Food, PWI-A = Personal Wellbeing Index, SF = Short-Form Health Survey,

ISCED = International Standard Classification of Education, Audit-C = Alcohol use disorders identification test – Consumption

^*^ p < 0.05,

^**^ p < 0.01,

^***^ p < 0.001

^1^ Model 1 without and model 2 with consideration of protective factors at KiGGS baseline survey

^2^ All models adjusted for age, gender, socioeconomic status, migration background and externalising problems

^3^ OR for categorical outcomes (binary logistic regression), RRR for categorical outcomes (multinomial logistic regression), B for metric outcomes (linear regression), negative coefficients indicate an opposite, positive coefficients an concordant association of the predictor with the respective outcome

^4^ With adjustment for protective factors

^5^ Models additionally adjusted for quadratic age term

^6^ Models additionally adjusted for fixed partnership

^7^ Lower case number due to filtering in the questionnaire

**Table 2:** Predicting developmental outcomes in young adulthood (age 21 to 31 years) in the presence of externalising problems in childhood or adolescence using linear and logistic regression models^1^ Source: KiGGS baseline survey (2003–2006), KiGGS Wave 2 (2014–2017)

Outcome in young adulthood^2^	Coefficient for externalising problems in Model 1^3^	Coefficient for externalising problems in Model 2^3^, ^4^
**Mental health**		
General mental health (MHI-5) (n=3,449)	B=-3.71^**^	B=-2.75^*^
Depressive Symptoms (PHQ-8) (n=3,456)	B=1.05^***^	B=0.79^**^
Panic disorder (PHQ panic) (n=3,421)	OR=1.08	OR=1.04
Eating disorder symptoms (SCOFF) (n=3,481)	OR=1.43^**^	OR=1.31^*^
**Life satisfaction and quality of life**		
General life satisfaction (PWI-A) (n=3,455)	B=-4.01^**^	B=-2.48
Physical quality of life (SF-8) (n=3,480)	B=-1.63^**^	B=-1.36^*^
Psychological quality of life (SF-8) (n=3,480)	B=-0.75	B=-1.25
**Education status (ISCED)**		
Low vs. medium (n=3,463)	RRR=2.53^***^	OR=2.37^**^
High vs. medium (n=3,463)	RRR=0.68^*^	OR=0.68^*^
**Substance use**		
Risky alcohol consumption (Audit-C)^5^ (n=3,479)	B=0.18	B=0.13
Smoking (n=3,492)	OR=2.50^***^	OR=2.39^***^
**Partnership, sexual and reproductive health**		
Permanent partnership (n=3,491)	OR=1.13	OR=1.13
Age of first sexual intercourse (n=3,192)	B=-0.71^***^	B=-0.71^***^
Number of sexual partners (n=3,206)	B=0.36^*^	B=0.34
General vs. occasional condom use^5^, ^6^ (n=3,177)	RRR=0.95	OR=1.00
No vs. occasional condom use^5^, ^6^ (n=3,177)	RRR=0.96	OR=0.98
Risk for unplanned children (n=340)^7^	OR=0.38^*^	OR=0.33^*^

B = Beta coefficient, OR = Odds Ratio, RRR = Relative Risk Ratio, MHI = Mental Health Inventory, PHQ = Patient Health Questionnaire,

SCOFF = Sick, Control, One, Fat, Food, PWI-A = Personal Wellbeing Index, SF = Short-Form Health Survey,

ISCED = International Standard Classification of Education, Audit-C = Alcohol use disorders identification test – Consumption

^*^ p < 0.05,

^**^ p < 0.01,

^***^ p < 0.001

^1^ Model 1 without and model 2 with consideration of protective factors at KiGGS baseline survey

^2^ All models adjusted for age, gender, socioeconomic status and migration background and internalising problems

^3^ OR for categorical outcomes (binary logistic regression), RRR for categorical outcomes (multinomial logistic regression), B for metric outcomes (linear regression), negative coefficients indicate an opposite, positive coefficients indicate a same-sense association of the predictor with the respective outcome

^4^ Adjusted for protective factors

^5^ Models additionally adjusted for quadratic age term

^6^ Models additionally adjusted for steady partnership and number of sexual partners

^7^ Lower case number due to filtering in the questionnaire
